# Pharmacogenetics in the Management of Coumarin Anticoagulant Therapy: The Way Forward or an Expensive Diversion?

**DOI:** 10.1371/journal.pmed.0020342

**Published:** 2005-10-25

**Authors:** Mike Greaves

## Abstract

Greaves discusses a new study in *PLoS Medicine* that takes us closer to being able to adopt a pharmacogenetic approach to reduce bleeding risk from coumarin therapy.

Warfarin and related coumarin-based anticoagulants are the mainstay of pharmacological management for the long-term prevention of thromboembolism. It is estimated that over 1 million residents in the United Kingdom take warfarin; in excess of 20 million prescriptions for the anticoagulant are dispensed each year in the United States of America. Despite recent advances in the development of novel, alternative, oral antithrombotics it is likely that coumarins will be used widely for at least the next decade.

## Mechanism of Action

Coumarins inhibit the posttranslational carboxylation of glutamate residues on proteins dependent on vitamin K, including coagulation factors II, VII, IX, and X. This carboxylation process is essential for their biological activity and requires the reduced form of vitamin K. Coumarins inhibit vitamin K epoxide reductase, the enzyme responsible for the recycling of vitamin K. The vitamin K epoxide reductase complex 1 (VKORC1) gene has been identified only recently [1] and this has permitted study of the effect of polymorphisms on sensitivity to coumarins.

The metabolism of warfarin depends principally upon the hepatic microsomal enzyme P450 2C9 (Cyp2C9), which catalyses degradation of the more potent S enantiomer to inactive metabolites.

## Variations in the Anticoagulant Effect

Coumarins have very few side effects. However, by far the most common unwanted effect, abnormal bleeding, may be lethal. Overall, the rate of life-threatening bleeding is around two per 100 patient years and more minor bleeding is common [2]. This is despite the widespread adoption of the International Normalised Ratio (INR) as the method for standardisation of the prothrombin time, the coagulation assay used to measure the anticoagulant effect of warfarin.

Individual patients on warfarin are within the target INR range for only 50%–70% of the time.

For most clinical indications, dosing is aimed at achieving a target INR of 2.5 (range 2.0–3.0), which represents a level of anticoagulation associated with an optimal relationship between antithrombotic efficacy and bleeding risk. However, even with the best available management, individual patients on warfarin are within the target INR range for only 50%–70% of the time, on average. Although the reasons for this imprecision of dose response are often not immediately apparent, important contributing factors include co-medication with interacting drugs [3] and incomplete adherence. In addition, the dietary content of vitamin K has a measurable effect on the INR in patients taking warfarin [4].

## A Genetic Component to Coumarin Sensitivity

In addition to this variation in dose-response within individuals, the inter-individual dose range required to achieve and maintain the target INR is exceptionally wide—for example, in the case of warfarin, a maintenance dose of 1 to >10 mg daily. Population studies suggested a genetic component to coumarin sensitivity; for example, people of Chinese origin are more sensitive, and African Americans less sensitive, than those of European ancestry [5,6]. This suggestion has now been confirmed; a significant proportion of the variation in warfarin sensitivity, including the variation between ethnic groups, can be accounted for by polymorphisms within the recently identified VKORC1 gene that influence transcriptional regulation [7]. This finding complements the earlier observation that variations in the rate of coumarin metabolism, due to the functional effects of polymorphisms in Cyp2C9, also contribute to individual coumarin sensitivity [8], as may polymorphisms in the genes for coagulation factors II and VII [9].

These findings raise the question of whether advances in understanding of the pharmacogenetics of oral anticoagulant therapy could beneficially influence clinical practice and patient safety by facilitating individualised coumarin dosing—hence reducing bleeding event rates. Answers are just beginning to emerge. In a retrospective study, Higashi et al. found that variant alleles of Cyp2C9 are associated with increased risk of above average INRs and longer duration to achieve stable dosing (compared to wild-type) in patients treated with warfarin [10]. Furthermore, patients with a variant genotype had increased risk of serious or life-threatening bleeding, although numbers of events were small, dictating caution in interpretation.

## A New Study

Additional evidence of the potential for a pharmacogenetic approach to reduce bleeding risk from coumarin therapy is presented in the current issue of *PLoS Medicine* [11]. Reitsma and colleagues used a case-control study design to examine the effects of the C1173T polymorphism in intron 1 of the VKORC1 gene on dose requirement and occurrence of severe bleeding in subjects treated with the long-acting coumarin phenprocoumon or short-acting acenocoumarol. A significant effect of genotype on the dose required to achieve target INR was confirmed. In addition, among users of phenprocoumon, but not among users of acenocoumarol, carriers of at least one T allele appeared to have an increased risk of bleeding as well as greater sensitivity to coumarin. However, counterintuitively, the quality of INR control appeared to be better in subjects on acenocoumarol.

These intriguing findings, along with those of Higashi and colleagues, suggest that further prospective studies of the utility of a pharmacogenetic approach to improving the safety of oral anticoagulation with warfarin are justified. Such studies will of necessity be large and should include cost-effectiveness analyses. They should be implemented, but the question arises whether their importance may be undermined by the licensing for long-term use of alternative, novel oral anticoagulants that have much more predictable dose-response characteristics, such as direct thrombin inhibitors.

## Abbreviations

Cyp2C9: hepatic microsomal enzyme P450 2C9
INR: International Normalised Ratio
VKORC1: vitamin K epoxide reductase complex 1
